# Nutrients and the Pancreas: An Epigenetic Perspective

**DOI:** 10.3390/nu9030283

**Published:** 2017-03-15

**Authors:** Andee Weisbeck, Rick J. Jansen

**Affiliations:** Department of Public Health, North Dakota State University, Fargo, ND 58102, USA; andee.weisbeck@ndsu.edu

**Keywords:** pancreatic cancer, epigenetics, nutrition

## Abstract

Pancreatic cancer is the fourth most common cause of cancer-related deaths with a dismal average five-year survival rate of six percent. Substitutional progress has been made in understanding how pancreatic cancer develops and progresses. Evidence is mounting which demonstrates that diet and nutrition are key factors in carcinogenesis. In particular, diets low in folate and high in fruits, vegetables, red/processed meat, and saturated fat have been identified as pancreatic cancer risk factors with a proposed mechanism involving epigenetic modifications or gene regulation. We review the current literature assessing the correlation between diet, epigenetics, and pancreatic cancer.

## 1. Introduction

The pancreas is important in transforming the food we eat into fuel for our body. The pancreas is made up of glandular tissue and a ductal system [1]. There are two different types of glands contained within the pancreas: exocrine and endocrine. The exocrine glands of the pancreas produce enzymes essential to digestion. Enzymes include trypsin, chymotrypsin, amylase, and lipase. Trypsin and chymotrypsin are involved in digesting proteins [1]. Amylase helps in the digestion of carbohydrate, and lipase works to break down fats. Pancreatic stellate cells residue within the exocrine areas of the pancreas and are responsible for aiding in tissue repair [2].

The endocrine portion of the pancreas contains islet cells referred to as islets of Langerhans. The islets of Langerhans produce and secrete hormones into the bloodstream and is directly responsible for maintaining homeostasis [3,4]. The islets of Langerhans contains four types of cells, each which secretes different hormones: alpha cells secrete glucagon, beta cells store and secrete insulin, delta cells secrete somatostatin, gamma cells secrete pancreatic polypeptide [2]. 

When there are insults or modifications to these normal functions, many different types of diseases can develop. Diabetes is the most common disorder of the pancreas and has several forms. Two forms of type 1 diabetes have been identified: type 1B has no known cause and is much less frequent and causes varying degrees of insulin deficiency, while type 1A is a result of the cell-mediated autoimmune attack on beta cells [5,6]. Type 1 diabetes occurs when the body fails to produce enough insulin to appropriately handle glucose and is characterized by elevated blood sugar levels, and lymphocytic penetration into the islets of Langerhans leading to the autoimmune response of T cells destroying beta cells [3,4,7] and subsequent inflammation [5,7].

Type 2 diabetes is complex and is characterized by the development of defects in insulin secretion and action, and generally accompanies weight gain, inactivity, and aging [6,8,9]. In type 2 diabetes, the pancreas produces insulin, however, the cells in the body cannot respond to insulin properly; this also referred to as insulin resistance [10]. The pancreatic islets respond to insulin resistance by increasing their cell mass and the secretion of insulin [9]. As a result, an excessive amount of glucose builds up in the blood stream rather than being stored for energy. As a result of insulin resistance, the pancreas can lose its ability to produce insulin because of beta cell hyperactivity leading to dysfunction [11]. High levels of blood glucose, over time, leads to damaged nerves and blood vessels. The damage to the nerves and blood vessels causes further complications with other organs of the body [12]. 

Lastly, type 3 diabetes mellitus or form type 3C tends to be underestimated and underreported. The endocrinopathy of type 3 diabetes is complex due to the presence of comorbidities including maldigestion [12]. Underlying type 3C are exocrine pancreatic diseases such as acute and chronic pancreatitis, cystic fibrosis, hemochromatosis, fibrocalculous pancreatopathy, pancreatic trauma, pancreatectomy, pancreatic agenesis and pancreatic cancer [12]. However, it seems that chronic pancreatitis is the most common [12]. Clinical characteristics are alteration in glucose metabolism, insufficient function of the exocrine function, and impairment of the incretin system [12]. 

In the United States, over 500,000 hospital admissions and over 800,000 emergency room visits can be attributed to gastrointestinal, liver, and pancreatic diseases [13]. Severe inflammation of the pancreas is referred to as pancreatitis. Pancreatitis can be acute or chronic. Acute pancreatitis appears suddenly and lasts a few days, while chronic pancreatitis is inflammation that occurs over many years. The long lasting inflammation can lead to permeant damage of the pancreas. The inflammation occurs when the digestive enzymes, which become active once secreted into the small intestine, become activated while still in the pancreas [13]. The activation of the digestive enzymes causes irritation and damage to the cells that produce the enzymes [14]. 

Pancreatic cancer occurs less frequently than many other types of cancer, but is the fourth most common cause of cancer death. Pancreatic ductal adenocarcinoma is most commonly found in the exocrine portion of the pancreas [15] and is the most lethal common cancer because of its resistance to therapy and its propensity to metastasize early in the disease progression [16,17]. Pancreatic cancer more frequently affects elderly men than any other population [16,17,18,19]. According the American Cancer Society, the five-year survival rate from 2003 to 2009 for pancreatic cancer was six percent [20]. However, of those diagnosed, an overwhelming percentage (85%–95%) of the cancers of the pancreas are ductal adenocarcinomas, therefore we will use pancreatic cancer and pancreatic ductal adenocarcinoma interchangeably [15,17]. 

The exact causes of this cancer are still unknown. Established risk factors associated with pancreatic cancer include smoking, family history, chronic pancreatitis, obesity, diabetes mellitus, diets high in fat and meat and low in vegetables and folate [18,20,21,22]. Hereditary factors have been associated with a percentage of the cases with a set of genetic abnormalities and environmental factors. Activation through mutations of KRAS oncogene, inactivation of tumor-suppressor genes such as CDKN2A, TP53, SMAD4, and BRCA2, telomere shortening, gene amplification, and chromosomal loss are the most frequent genetic mutations [16,17,21,22,23,24]. In addition to the mutations in genes, epigenetic alterations in DNA methylation, histone modification, and non-coding RNA can change gene function in pancreatic cancers [23,24,25,26]; this topic will be further discussed below. 

To date, there have been significant and important advances in understanding the molecular biology of pancreatic cancer, which have improved the diagnosis, staging, and treatment for patients. However, little progress has been made in preventing disease onset or diagnosing patients in early stages. This review will focus on dietary factors associated with pancreatic ductal adenocarcinoma and the potential influence epigenetic factors play in these associations. 

## 2. Methodology 

**Figure 1:**
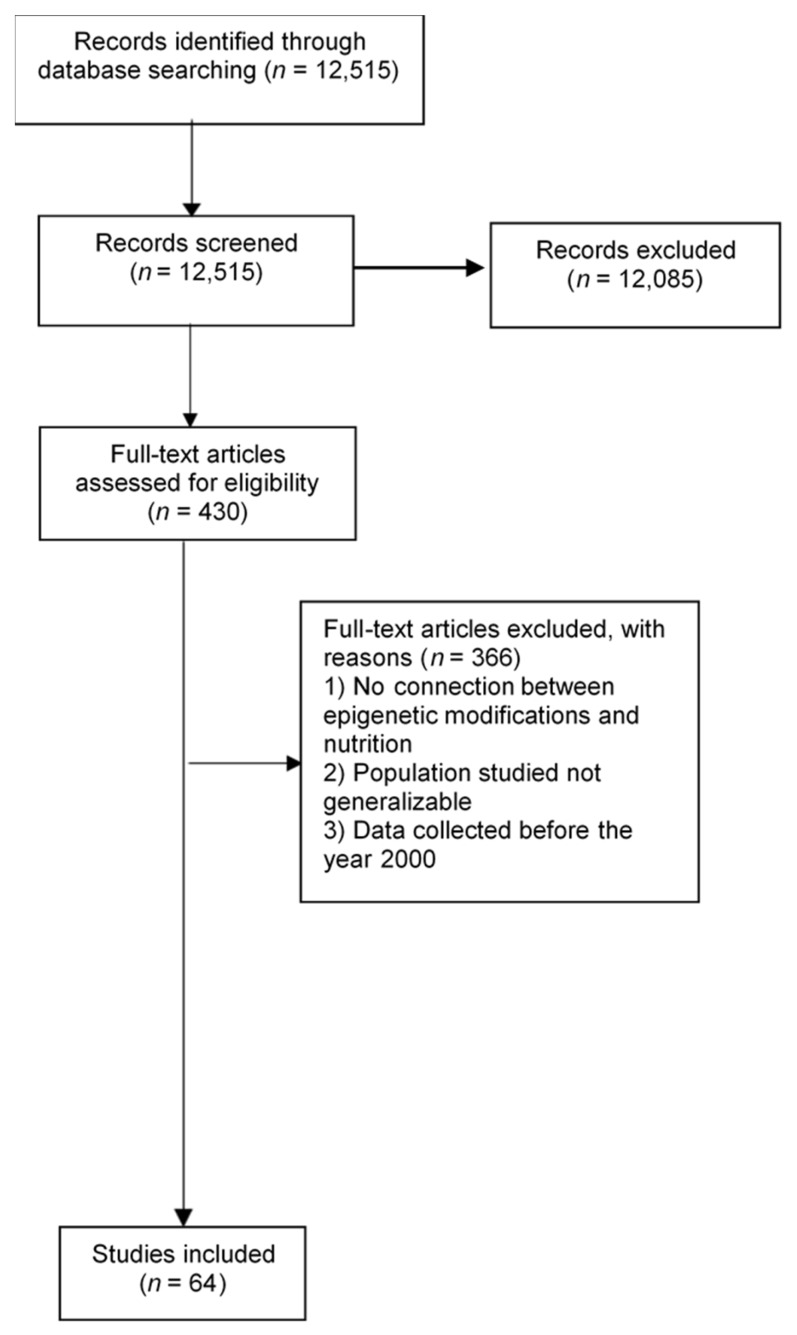
Flow diagram of included studies.

We conducted a search of the published scientific literature using Pubmed, SpringerLink, Google Scholar, EBSCO, Elsevier, and Wiley Online Library (with the search terms “pancreatic cancer” and “epigenetics” or “nutrition” or “diet” or “epigenetic modifications”). In these databases, topic searches were performed examining keywords, titles, and abstracts for selected terms. Reference lists of selected studies were also hand-searched. Our search was not restricted by language or date. Articles were screened through titles and abstracts; we reviewed full texts of articles that met prespecified inclusion criteria. We included systematic review articles, meta-analyses, and primary research articles. Figure 1 shows the results from the literature search and study selection.

Data was extracted from included studies. Extracted data included study goal, background information, nutrients associated with pancreatic cancer, and epigenetic mechanisms involved in pancreatic cancer development.

## 3. Evidence Linking Dietary Intake to the Pathogenesis of Pancreatic Cancer 

Pancreatic cancer is a rare and complex disease that takes over 10 years to develop. There are numerous difficulties when trying to ascertain retrospective dietary histories from patients including recall bias to misdiagnosis [27,28]. However, despite difficulties related to studying pancreatic cancer and diet, evidence from epidemiologic research strongly suggests diets high in fat and red meat, diets low in vegetables and certain fruits, and low folate consumption are associated with pancreatic cancer. Table 1 provides a summary of the dietary compounds and their link to pancreatic cancer.

### 3.1. Folate

The significant inverse relationship between dietary folate intake and pancreatic cancer risk is observed in most studies [27,28,29,30,31,32], but not all [33,34]. A meta-analysis [35] reported an inverse relationship between dietary folate intake and pancreatic cancer risk. Additionally, two other case-control studies and several cohort studies observed an inverse relationship [30,31,36,37,38]. The specific mechanism in which folate protects against pancreatic cancer is unknown, however a few are hypothesized. 

Folate donates dietary methyl groups involved in DNA synthesis and repair and DNA methylation and irregularities and these pathways may contribute to cancer [35]. Low folate or defective folate metabolism leads to methyl group deficiency. According to McCabe and Cadudill [32] deficiencies in folate and their methyl supplying ability can either independently or interactively reduce the potential for DNA methylation. This deficiency is thought to be linked to decreasing levels of *S*-adenosyl-methionine, reducing global DNA methylation, as well as reducing the conversion of uracil to thymidine, increasing DNA breaks causing genetic instability [25]. 

Folate deficiency can also cause alter expression for proto-oncogenes and tumor suppressor genes because of abnormal methylation [29]. The enzyme 5,10-methylenetetrahydrofolate reductase (MTHFR) is needed for folate metabolism. Genetic changes in this enzyme may result in an increased risk of pancreatic cancer because MTHFR works by directing folate metabolites towards the DNA methylation pathway [29]. MTHFR is also involved in DNA methylation and abnormal DNA methylation is associated with many types of cancer, including pancreatic [32]. 

### 3.2. Vegetables/Fruit

Several studies have assessed the association between pancreatic cancer and vegetable and fruit consumption. The World Cancer Research Fund/American Institute for Cancer Research Joint Committee determined fruits and vegetables have a protective effect against pancreatic cancer [39]. However, individual studies show inconsistent results. Potential reasons for these inconsistencies across studies include sample size, study type, and dietary assessment method.

A case-control study in the Czech Republic, found that a high consumption of citrus fruit as well as more than three portions of cooked vegetables per week had significant protective effects against pancreatic cancer [40]. Additional case-control studies completed in China and Italy observed a direct inverse relationship between vegetable consumption and pancreatic cancer [36,41]. However, a Canadian case-control demonstrated an increased intake of fresh fruit and cruciferous vegetables in men only was associated with a 49% reduction in risk [42]. 

The inverse relationship is attributed to the biological mechanisms related to cell signaling and cell regulation, and is likely due to antioxidant, antimutigentic, and antiproliferative properties associated with nutrients found in fruits in vegetables [43]. Other studies have associated the protective effect with relationship to diallyl disulfide and sulforaphone (present in cruciferous and Allium vegetables) to have the ability to inhibit histone deacetylase (HDACs) enzymes [44,45]. Evidence has shown these dietary components inhibit cell proliferation and stimulate apoptosis [40]. In addition, isothicyanates, an enzymatic product of a plant enzyme called myrosinase, has been shown in laboratory studies to inhibit the growth of human pancreatic cancer cell lines [27,44,46].

Several nutrient found in fruits and vegetables have been associated with pancreatic cancer prevention. Vitamin C, has been hypothesized to protect against cancer by preventing oxidative damage to DNA and polyunsaturated fatty acids [47]. As well, Vitamin C has been found to inhibit the formation of carcinogenic substances (*N*-nitrosation) in food and in the gastrointestinal tract, which may be involved in the development of cancer [47]. 

Phytochemicals found in cruciferous vegetables are also thought to be involved in cellar pathways, such as apoptosis, halting or preventing carcinogenesis [47]. Two important sulfur-containing phytochemicals glucosinolates (GS) and *S*-methyl cysteine sulfoxide (SMCSO) are present in cruciferous vegetables and appear to have anticarcinogenic properties [47]. 

### 3.3. Red/Processed Meat

Red and processed meat consumption has been shown to be related to pancreatic cancer. However, again the association is inconsistent. A meta-analysis conducted showing the relationship between red and processed meat consumption and increased risk of pancreatic cancer identified a significant association with red meat and increased risk of pancreatic cancer in men, and a significant positive association for processed meat [48]. Ghorbani et al. [49], found a 67% increased risk of pancreatic cancer with an increased consumption of red meat, specifically barbecued. This is consistent with the findings in Jiao et al. [50] stating that high cooking temperatures is related to increased amounts of *N*-(carboxymethyl)lysine (CML) advanced glycation end products (CML AGEs). CML AGEs possibly contribute to insulin resistance, oxidative stress, and chronic inflammation [42,43,44,45,46,47,48,49,50,51,52,53]. Moreover, CML AGEs are speculated to contribute to the development of pancreatic cancer by changing the tissue stroma environment [50]. Therefore, there is building evidence that the consumption of thermally processed red meat consumption contributes to pancreatic cancer development. This association between the consumption of red/processed meat and pancreatic cancer may also be explained by the mutagenic compounds produced during the cooking or preservation process [54]. Ghorbani et al. [49] associated the increased risk of pancreatic cancer to high amounts of heterocyclic amines (HCAs) and polycyclic aromatic hyrdocarbons (PHA) produced from barbecuing. Heterocyclic amines are formed when meat is cooked at high temperatures. While, polycyclic aromatic hydrocarbons are produced when meats are grilled or charcoal broiled [54]. Furthermore, several epidemiological studies have shown the positive association of grilled and barbecued meat with an increased risk of pancreatic cancer [55,56,57,58,59,60,61]. 

### 3.4. Saturated Fatty Acids

A limited number of studies have been conducted investigating the link between saturated fatty acids and pancreatic cancer, and the results are inconsistent. Saturated fatty acids are known to effect insulin secretion and insulin resistance, and although there is mounting evidence suggesting that insulin resistance is involved in the carcinogenesis of the pancreas, the mechanisms in which dietary fats effect insulin action is unclear [60]. Using the National Institutes of Health-AARP Diet and Health Study, Thiebaut et al. found a significant association between saturated fat intake and pancreatic cancer [61], while in a laboratory study, a diet rich in saturated fatty acids showed an increase in expression in a key regulator of beta-oxidation and was increased in pancreatic cancer cells [62]. As another mechanism, fatty acids may regulate cancer cells by modulating hypoxia-inducible factor-1 (HIF-1) which encode for proteins including glucose transporters and growth factors [62]. An analysis of a large cohort study of male smokers reported a significant positive association for pancreatic cancer and saturated fat intake [63]. Additionally, this study hypothesized the relationship to be due to the carcinogens, heterocyclic amines and polyaromatic hydrocarbons but may not be generalizable for nonsmokers. 

In addition, dietary fat was demonstrated by Matters et al. to induce growth and metastasis of pancreatic cancer [64]. The method in which fatty acids accelerates tumor growth is partial mediated by the interaction of the gastrointestinal peptide cholecystokinin, CCK, and its receptor. CCK is responsible for stimulating secretion of digestive enzymes from the pancreas, releasing bile from the gall bladder, and mediates satiety in the brain [64]. Thus, endogenous CCK and its receptor are important in tumor progression and metastasis of pancreatic cancer, particularly within the context of high fat consumption [64]. 

## 4. Epigenetic Modifications Related to Dietary Intake and Pancreatic Cancer Risk

In addition to the known genetic modifications, the role of epigenetic alterations has become more apparent in the development and progression of cancer. However, there is still limited evidence that demonstrates the associations in more than one study or in detail. Therefore, the scope of this section is restricted but summarizes the current knowledge and evidence available.

Epigenetics can be described as heritable changes in genes without having changes in DNA sequences [24]. DNA methylation, histone modification, and microRNA expression are known epigenetic mechanisms that affect gene expression and modifications have been associated with cancer [24]. Two main hypothesis currently exist around how dietary intake influences development and progression of pancreatic cancer: (1) certain dietary components affect pathways involved in insulin resistance and insulin insensitivity; and (2) dietary components reduce oxidative stress and inflammation by reducing DNA damage and mutation [27]. 

### 4.1. DNA Methylation

DNA methylation is the addition of a methyl group (CH3-) to the 5-carbon of cytosine residues. This mechanism is catalyzed by enzymes in the DNA methyltrasferase (DNMT) family. The major pattern of DNA methylation occurs in DNA stretches with a high CG nucleotide content (CpG islands) and are often located near promoter regions of genes [23,65]. 

In pancreatic cancer, epigenetic silencing is frequently observed with altered methylation of CG dinucleotides (CpG islands) occurring within the regulatory regions of tumor suppressor genes and critical homeostatic pathways [22]. DNA methylation patterns have been associated with carcinogenesis and are known to interfere with the gene stability and transcription [65]. While the overall effect of DNA methylation is to modify gene expression, it is also involved in maintaining a repressive chromatin state [32,66,67]. In addition, evidence suggests nutrients that supply and regenerate methyl groups, such as folate, influence patterns of DNA methylation [29,32].

Modifications to DNA methylation can either cause a hyper- or hypomethylated state within the cell. Hypermethylation is associated with over regulation and gene silencing by affecting the affinity of transcription factors [67]. CDKN2A/p16 was one of the first recognized tumor suppressor genes to undergo hypermethylation at the promoter region and lead to gene silencing in pancreatic cancer [23]. Hypomethylation has also been implicated in pancreatic adenocarcinomas. Hypomethylation is associated with increased chromosomal loss and genomic instability resulting in loss of regulation and promotion of genes and protein expression [23]. Folate deficiency has been associated with hypomethylation [23,25,32].

A pathway analysis involved in pancreatic ductal adenocarcinoma, revealed 25 pathways that are significantly influenced by DNA methylation [68]. Cell adhesion, hedgehog signaling, transforming growth factor-β (TGF-beta), integrin signaling, and WNT (Wingless/Int1)/Notchsignaling are well known key cancer signaling pathways that were shown to be aberrantly methylated in pancreatic cancer [68]. Additionally, stellate cell activation and axon guidance were significantly affected by DNA methylation in adenocarcinomas [68]. Also, DNA methylation has been implicated in the development of diabetes, influencing islets, beta and alpha cells, contributing to malignant cell transformation [26]. 

### 4.2. Histone Modification

Histones determine the structure of chromatin and the changes to this structure influence gene expression. The histones can undergo post-translational modifications, and the modifications can facilitate or hinder DNA repair proteins and transcription factors [24,44]. Histone acetylation and methylation represent the two epigenetic modifications for which current data exists in the context of pancreatic cancer [24]. However, few studies have examined the specific genes regulated by histone modifications in cancers of the pancreas.

Modification through acetylation of histone tails is controlled by two families of enzymes: histone acetyltransferases (HATs), which transfer an acetyl group, and histone deacetulases (HDACs), which remove acetyl groups [44,69]. These enzymes are essential in cellar functions including: chromosome remodeling, gene transcription, and cell proliferation [69]. Changes in histone methylation have been shown to be associated with cancer susceptibility [44]. 

Within human pancreatic islets cells, histone modifications have been associated with activation or repression of genes [70]. Genes of the mucin family have been shown to be over expressed in pancreatic cancers due to histone alterations [25,71,72]. In other studies, CDKN1A—a tumor suppressor gene regulated by p53 mechanisms encoding for p21—was shown to be acetylated on H3 and H4, which may account for cell cycle arrest induced by diallyl disulfide, which is found in vegetables [44,73]. 

### 4.3. miRNA

MicroRNAs (miRNA) are involved in the regulation of biological processes in gene silencing pathways [74,75]. These molecules are classified into either oncogenes or tumor suppressors depending on their targets, thus binding to oncogenes or tumor suppressor genes [75]. A handful of microRNAs were shown to have changes in expression pancreatic cancer [17,22,24,69,76,77,78]. For example, Torrisani et al. [78] found that let-7 miRNA, a tumor suppressor, is expressed in normal pancreatic cells, but significantly downregulated in pancreatic ductal adenocarcinoma samples. Multiple cellar functions were found to be inhibited by let-7 miRNA, including Kras expression [75]. Other miRNAs were found to effect pathways involved in cell cycle and proliferation, DNA repair, apoptosis, invasivity, and metastasis in cancers of the pancreas [17,22,75,79,80]. Several miRNAs have also been implicated in β-cell dysfunction, thereby affecting insulin regulation in association with type 2 diabetes [26,68,76,81] and potentially pancreatic cancer. 

### 4.4. Long Non-Coding RNA

Long non-coding RNA (lncRNA) are RNA molecules that are longer than 200 nucleotides, but are not translated into a protein. Only a few lncRNAs have been characterized, but recent studies have identified that certain lncRNA are specifically associated with certain cancers, such as breast, prostate, liver, and colorectal [82,83]. Expression levels of lncRNAs are linked to recurrence, metastasis, and prognosis of these cancers [82,84]. lncRNAs have been shown to have regulatory functions related to epigenetics, transcription, cell growth and apoptosis [82,84,85,86]. 

A few lncRNAs (HOTAIR, HULC, MALAT1, HOTTIP) have been found to be associated with pancreatic cancer [84,86,87,88,89,90]. Research by Chang et al. demonstrated the ability of HOTAIR to function as scaffolds to assemble histone modification complexes [87]. HOTAIR regulates function through epigenetic modifications and exhibits pro-oncogenic activity [87,91]. HULCs role and function in pancreatic cancer, however, is largely unknown. In a study by Peng and Gao, HULC was upregulated in pancreatic cancer tissue compared to surrounding normal tissue [84]. HULC was also associated with tumor size, vascular invasion, and lymph node metastasis [84]. MALAT1 is highly expressed in pancreatic cancer and is thought to enhance stem cell-like phenotypes [89] and its expression has been significantly correlated with the malignant statues of pancreatic cancer patients [86]. HOTTIP was observed to have pro-oncogenic functions in pancreatic cancer, similar to that of HOTAIR, although they elicit their effects through different pathways, leading to the expression of different sets of genes [88]. Growing evidence suggest lncRNAs participate in all stages of tumor initiation and development [85]. However, the molecular mechanisms in which lncRNAs act to influence pancreatic cancer is still largely unknown and warrants further research [85].

## 5. Conclusions

The purpose of this study was to review the current evidence surrounding the role of diet on the development and progression of pancreatic cancer. Although studies are inconsistent, mainly due to study type and the nature of the disease, there is significant evidence that certain dietary items are associated with pancreatic cancer. There is still much research needed to explore the connection between epigenetic modification and dietary intake and their association with pancreatic cancer. Many gaps exist in assessing this direct mechanistic relationship. Although there is significant hypothetical evidence for a role of diet to modify epigenetic mechanism and cause the development or progression of pancreatic cancer, more research is required to gain a better understanding of the potential molecular mechanisms behind pancreatic cancer development and progression.

## Figures and Tables

**Table 1:** Summary of dietary components and their link to pancreatic cancer.

Nutrient/By-Product	Epigenetic Relation	Link to Cancer
Folate	Donation of methyl groups	Reduced DNA methylation, increased DNA breaks, altered expression of proto-oncogenes and tumor suppressor genes
Diallyl disulfide	Inhibit histone deacetylase (HDACs) enzymes	Inhibit cell proliferation and stimulate apoptosis
Sulforaphone	Inhibit histone deacetylase (HDACs) enzymes	Inhibit cell proliferation and stimulate apoptosis
Vitamin C	Prevents oxidative damage to DNA and polyunsaturated fatty acids, formation or carcinogenic substances (*N*-nitrosation)	Anticarcinogenic properties
*N*-(carboxymethyl) lysine (CML) advanced glycation end products	Mechanism unknown	Mutagenic compounds produced during cooking or preservation process
Heterocyclic amines (HCAs)	Mechanism unknown	Mutagenic
Polycyclic aromatic hydrocarbons (PHA)	Mechanism unknown	Mutagenic
Saturated fatty acids	Modulating hypoxia-inducible factor-1 (HIF-1), method by which fatty acids accelerate tumor growth is mediated by gastrointestinal peptide cholecystokinin (CCK) and its interaction with its receptor	HIF-1 encodes for proteins including glucose transporters and growth factors, CCK and its receptor are important in tumor progression and metastasis of pancreatic cancer
Phytochemicals	Inhibits the initiation of chemical carcinogenesis	Anticarcinogenic properties
